# Urbanization in India: Population and Urban Classification Grids for 2011

**DOI:** 10.3390/data4010035

**Published:** 2019-02-26

**Authors:** Deborah Balk, Mark R. Montgomery, Hasim Engin, Natalie Lin, Elizabeth Major, Bryan Jones

**Affiliations:** 1 CUNY Institute for Demographic Research, City University of New York, New York, NY 10010, USA; 2 Baruch College Marxe School of Public and International Affairs, City University of New York, New York, NY 10017, USA; 3 Population Council, New York, NY 10017, USA; 4 Stony Brook University, New York, NY 11794, USA

**Keywords:** India, urbanization, population, census, built-up area, Global Human Settlement Layer, grid, raster

## Abstract

India is the world’s most populous country, yet also one of the least urban. It has long been known that India’s official estimates of urban percentages conflict with estimates derived from alternative conceptions of urbanization. To date, however, the detailed spatial and settlement boundary data needed to analyze and reconcile these differences have not been available. This paper presents gridded estimates of population at a resolution of 1 km along with two spatial renderings of urban areas—one based on the official tabulations of population and settlement types (i.e., statutory towns, outgrowths, and census towns) and the other on remotely-sensed measures of built-up land derived from the Global Human Settlement Layer. We also cross-classified the census data and the remotely-sensed data to construct a hybrid representation of the continuum of urban settlement. In their spatial detail, these materials go well beyond what has previously been available in the public domain, and thereby provide an empirical basis for comparison among competing conceptual models of urbanization.

## 1. Introduction

India, the world’s most populous country, is also one of the least urban. At the time of the most recent census in 2011, 31% of the country’s population lived in urban areas according to the official statistics [1]. Yet by other accounts, this 31% figure is far too low [2–4]. Such disagreements stem from different conceptual models of urbanization—the higher percentages are derived from a “statistical” perspective in which urban-ness is defined in terms of population density, areal contiguity, and the total population of sufficiently dense contiguous areas. This approach stands in contrast to the official classifications for India, which blend the statistical perspective with an alternative view that takes the legal boundaries of urban jurisdictions into account. Both perspectives have merit; but it has proven difficult to reconcile them because the data needed to do so have not been available in the public domain.

This paper places into the public domain two detailed sets of gridded estimates of urban and rural areas at 1 km (or finer) resolution. One set is based on India’s official criteria, providing a spatial representation of urban and rural communities as these are officially defined. The second set of estimates is informed by the statistical perspective on urbanization, drawing on approaches that integrate disparate data to indicate urban population [5]; these estimates extend the official criteria via remotely-sensed measures of the density of structures, obtained from the Landsat imagery processed by the Global Human Settlement Layer research team [6]. Taken in combination, our estimates provide what has long been lacking: an empirical basis for a rigorous comparison of competing urban definitions. We have made available two 2011 snapshots capturing a moment in India’s upward trajectory of urbanization, which in the coming decades will have wide-ranging consequences for human well-being and the natural environment [7–11]. To accompany these two urban classification grids, we also produce a grid of population using the same finely-resolved spatial units—not previously used as inputs in any other spatial population dataset—so that urban location and population may be examined together.

## 2. Data Description

Before describing the data collection on population and urban classifications in India that we have produced and disseminated with this article, we should first explain the empirical ingredients.

### 2.1. Input Data

Three types of data were needed to generate high-resolution grids of India’s urban areas and population: Settlement-level demographic data from the 2011 population census; spatial boundaries that delineate these settlements; and remotely-sensed data on built-up area. Each is described in turn.

#### 2.1.1. Population Census Abstracts

In a welcome departure from previous practice, India’s Office of the Registrar General and Census Commissioner has placed into the public domain a very large collection of detailed, settlement-specific tabulations of 2011 population census data. For the purposes of this research, the key tabulations come in the form of what are termed primary census abstracts (PCAs)—they cover places ranging in size from tiny rural villages to small- and medium-sized towns and upward to the largest of India’s municipalities, providing information on the population of each settlement, its number of households, and selected additional characteristics. Complementary spatial data—to be described below—are available for a total of 4041 legally-constituted urban areas (*statutory towns*), 3893 so-called *census towns*, and 640,930 rural *villages*. The statutory towns are further subdivided into *wards*, with an abstract produced for each ward. Some of these wards are additionally designated as *outgrowths*.

The four urban categories—statutory towns, wards, outgrowths, and census towns—require a few words of explanation. Statutory towns are governed by one of the many forms of urban local governmental authority that exist in India, with the Constitution giving considerable latitude to state governors in decisions about whether and what type of authority to establish. The legal basis is set out in the Constitution of India PART IXA-243Q, as follows:

“Constitution of Municipalities. (1) There shall be constituted in every State, (a) a Nagar Panchayat (by whatever name called) for a transitional area, that is to say, an area in transition from a rural area to an urban area; (b) a Municipal Council for a smaller urban area; and (c) a Municipal Corporation for a larger urban area, in accordance with the provisions of this Part: Provided that a Municipality under this clause may not be constituted in such urban area or part thereof as the Governor may, having regard to the size of the area and the municipal services being provided or proposed to be provided by an industrial establishment in that area and such other factors as he may deem fit, by public notification, specify to be an industrial township. (2) In this article, ‘a transitional area’, ‘a smaller urban area’ or ‘a larger urban area’ means such area as the Governor may, having regard to the population of the area, the density of the population therein, the revenue generated for local administration, the percentage of employment in non-agricultural activities, the economic importance or such other factors as he may deem fit, specify by public notification for the purposes of this Part.”

Wards are electoral units that are overseen by statutory-urban governing bodies. There is no automatic rule by which statutory towns and their constituent wards come into being on the basis of well-defined demographic and economic criteria. Indeed, a notable and much commented-upon feature of Indian urbanization has to do with the reluctance of some states to allow their large, urban-like villages to be legally declared urban. The public finance problem is that an urban authority may not be eligible for development funds that are earmarked for rural areas, and depending on circumstance, state governors may be unpersuaded of the potential for obtaining commensurate urban development funds [2–4]. These dueling political–economy considerations may well combine to produce under-estimates of India’s urban percentages, an issue that we will investigate in what follows.

Among all the wards of a statutory town, some can be designated as outgrowths, these being units which hold a type of dual status. An outgrowth is an area of high-density, arguably urban settlement that is spatially adjacent to a statutory town, and which would thus seem to be poised on the threshold of becoming legally urban. However, outgrowths are in fact governed by rural authorities. The ambiguous status of outgrowths is signified in their PCA identifier codes: outgrowths are assigned both a village code and a code defining the outgrowth as a ward of the statutory town. In India’s tabulations of urban population, outgrowths are treated as urban.

Much like outgrowths, census towns are legally rural settlements, but they are designated as urban for the purposes of an upcoming census and grouped with statutory urban areas in the official post-census tabulations. The census-town designation emerges in the course of discussions between census authorities and state government officials in the lead-up to each new census [12–14]. There are specific demographic and socioeconomic criteria that are meant to guide the discussions, but these criteria are evaluated on the basis of data gathered in the previous census, a practice that leaves ample room for misunderstandings of local trends and variations in judgement [14]. Also, there is no requirement that once classified as urban in this specialized way for a given census, a census town must remain so classified for the next decennial census. It seems that the state-specific discussions effectively begin anew in each census round. The census town–statutory town distinction has been in place for many decades, and India’s system of identifier codes for settlements has long distinguished the two.

Our calculations reveal how important census-town designations are, for example, to the state of Kerala’s overall percentage urban. The 2011 Census put the urban percentage of India as a whole at 31.1 percent, with census towns accounting for only 4.2 percentage points of the total. In Kerala, however, roughly 50.8 percent of the population is urban, a total that is well above the all-India average, with census towns accounting for almost 29 points of this total. Indeed, had the census towns of Kerala been ignored, only 21.9 percent of the state’s residents would have been counted as urban. Since the status of “census town” holds only for a given census, these towns can transition from rural village to census-urban status and then back, or alternatively can go on to become statutory urban, a complication that spawns confusion about the longer-term meaning of India’s reported urban percentages and which obscures the true pace of the country’s urbanization.

#### 2.1.2. Boundary Data

Although the PCA settlement-level tabulations have been placed in the public domain, the government of India has not released digital records of settlement boundaries. Boundary data must be purchased from third-party vendors, who prohibit their redistribution. We use the proprietary “Village Map” data products (one for each state or union territory, in the WGS 1984 geographic projection) from ML Infomap LLC to provide the vector settlement boundary input for our new grids. (It is impossible to reconstitute the original boundaries from our new, gridded data products.) According to the ML Infomap metadata, its “coastal boundaries were aligned with imagery, [with] no gaps in polygon or no topological errors” and assure an “accuracy of the boundaries to 30–50 m”. These spatial data include the key identifiers (indicating state, district, subdistrict, and settlement) which (with some exceptions) allow the boundaries of each settlement to be linked to the corresponding PCA record and thereby to the full set of PCA demographic indicators. In total, over 650,000 spatial units have been used to construct the data collection provided in this research, which represents more than a 100-fold increase in the input resolution over the best publicly-available alternative, The Gridded Population of the World, Version 4. (GPWv.4) [15]. Although the settlement boundaries are primarily rendered as vector polygons, in some less populous states, settlements could only be represented in terms of point locations. (Implications of this for our gridding method are discussed below).

**Figure 1.**
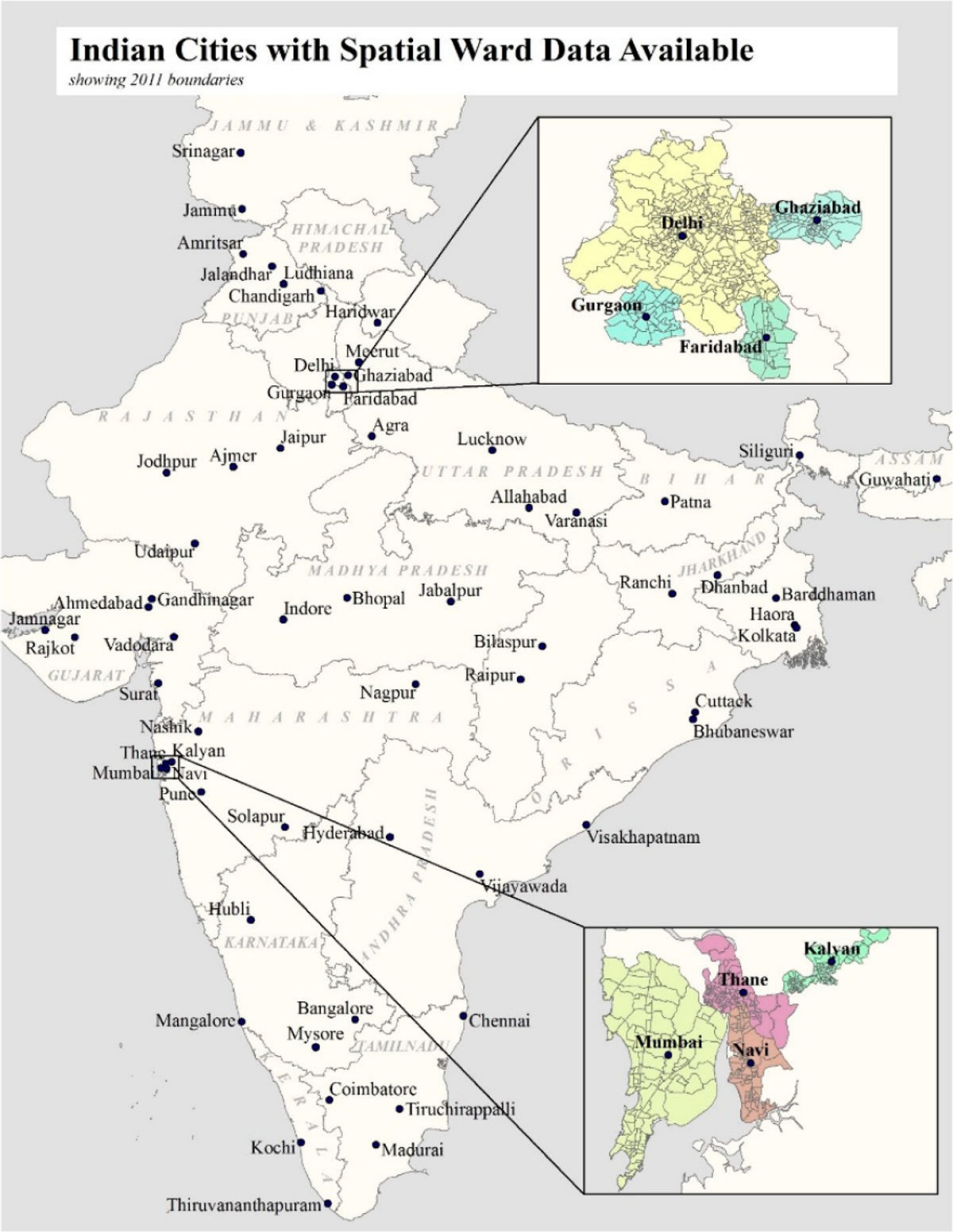
Data for India with indications for which cities we had spatial ward-level data.

Table 1 below lists, by state as well as for India as a whole, the spatial inputs that we have used in this research. For statutory towns, census towns, and villages the table gives the number of whole settlements available in the spatial data. Many of these settlements are sub-divided into components that are specific to administrative district or subdistrict, but here we report only the total number of settlements. Spatial records for outgrowths are also available for the whole of India. However, ward-level spatial data are only available from ML Infomap as separate proprietary products. For a subset of 62 cities, including all cities with a population above 1 million, we collected additional ward-level spatial information, as shown in Figure 1. Indian cities are internally organized in a variety of ways, and as the map in Figure 1 demonstrates, the ratio of settlement-level to ward-level units varies from one city to the next. Mumbai, a city of over 12 million inhabitants, consists of two distinct settlements and 97 wards. Navi Mumbai, a city of just over 1 million in the Greater Mumbai region, is 1 settlement with 89 wards, giving it twice the ratio of wards to settlements as Mumbai for about one-tenth of the population. The ward-level detail reveals population and density variations within cities and helps to identify uninhabited areas (e.g., large urban parks or reserved land) that would otherwise skew the density estimates. The distribution of population within cities is a very important factor in assessing population exposed to spatially-specific environmental risks, for example, flooding.

The original spatial data from ML Infomap were thoroughly cleaned and subjected to multiple rounds of topological correction. In the course of cross-validating the ML Infomap spatial data with census information and open-source settlement information, we uncovered numerous although generally minor flaws in these proprietary data. As described below, we made alterations only where necessary to achieve a match to the PCA records, and only if authoritative boundary information lent support to the changes. (Many additional alterations could have made to the original boundary data but were not due to inconsistencies and deficits in authoritative boundary records in India.) Furthermore, because district-level boundary data from ML Infomap are used in other 1-km gridded data products (such as GPW v.4), we decided to make the fewest alterations possible, systematically adjusting only the settlement polygons that were misaligned with district or state borders.

In particular, when we attempted to merge ward and outgrowth data from the PCAs with their corresponding ML Infomap spatial boundaries, it became clear that some wards and outgrowths (as well as some census towns), were either omitted from, or clearly misrepresented by, the spatial data. To achieve adequate linkages, we were required to split and merge ward polygons, and needed to draw new polygons where the omitted units could be identified with confidence. Publicly available data, including the *District Census Handbooks* and *Atlases* [16,17] of the Indian Census, resources on Open Street Map, and the ESRI base-map and Google were used in this data-cleaning process. In total, over 200 new polygons were either created or substantially altered. These corrections made it possible to achieve a match with the PCA data for nearly all of India’s settlements and outgrowths. Of the about 1036 outgrowths identified in the PCAs, only seven have not yet been located in the spatial boundaries data. The populations of these missing outgrowths are known, as are the statutory towns to which they are adjacent; only their precise spatial locations are yet to be established. (Additionally, a small number (*n* = 57) of outgrowths, were aggregated in the ML Infomap spatial data. Maps provided in the Census *Atlases* and *District Census Handbooks* were not sufficiently informative to identify the individual outgrowth boundaries, leaving us no option but to aggregate, the PCA records to match the ML Infomap spatial units (*n* = 11).)

The ward-level spatial data supplied by ML Infomap for Delhi, New Delhi, and certain cities in Andhra Pradesh do not always respect the fine partitions by administrative sub-district that are found in the PCA census records. We hope to resolve this problem in future research, but in the present data collection we have chosen to represent such problematic statutory towns spatially by their outer settlement boundaries and use whole-settlement PCA summaries to account for their populations.

#### 2.1.3. Global Human Settlement Layer (GHSL) Data

Where spatial boundaries for urban settlements are lacking, out of date, or subject to conflicting interpretations, satellite data can be invaluable in identifying areas of human activity, whether by indicating built-structures or night-time lights. Such data have been used in recent decades to serve as proxies for urban areas [18]. Here we employ the Global Human Settlement Layer (GHSL) produced by the Joint Research Center (JRC) of the European Commission. These data represent a new generation of global built-up land data products, ranging over 40 years of historic change (1975, 1990, 2000, and 2014; these are the ‘epochs’ or year on which the satellite observations were made) at fine spatial resolution (approximately 30 m in original form, aggregated to 250 m). The GHSL rasters were released in World Molleweide projection (datum: D_WGS_1984). Our research makes use of the 2014 built-up areas for India, as other studies have also done [19,20] rather than interpolating data from 2000–2014 to match the 2011 census, despite the possibility that additional built-up areas may have emerged in the three years since the census was conducted.

In their original form, the GHSL data are binary, indicating either the presence or absence of a built structure in each 30 m grid cell [6,18,21–24]. A cell is coded as built-up if it overlaps with a built structure or impervious surface (but not roads). In the version of GHSL used here, the 30 m cells were aggregated to a resolution of 250 m and assigned the proportion of built-up land as the raster value. Recent research has generally confirmed acceptable levels of accuracy of the GHSL except perhaps in very thinly settled rural regions; for details, see studies of omission errors in the rural United States [20,24]. While similar validation studies for India have not been undertaken, Corbane and colleagues [18] report that errors of omission in the newest GHSL product—the one we use here that is based on Sentinel-1 data in addition to Landsat imagery—are substantially reduced in Asia from the first generation (Landsat-only version).

### 2.2. Output Data

All output are raster-format grids at a spatial resolution of 1 km, with the exception of the urban cross-classification grid, *Census + GHSL*, which we produce at a 250 m resolution in line with the 250 m GHSL data. Table 2 lists the datasets that we have constructed. These are disseminated by state, with the files identified by use of a two-character state-code at the start of all file names. A list of available datasets is found in Table 2.

**Figure 2.**
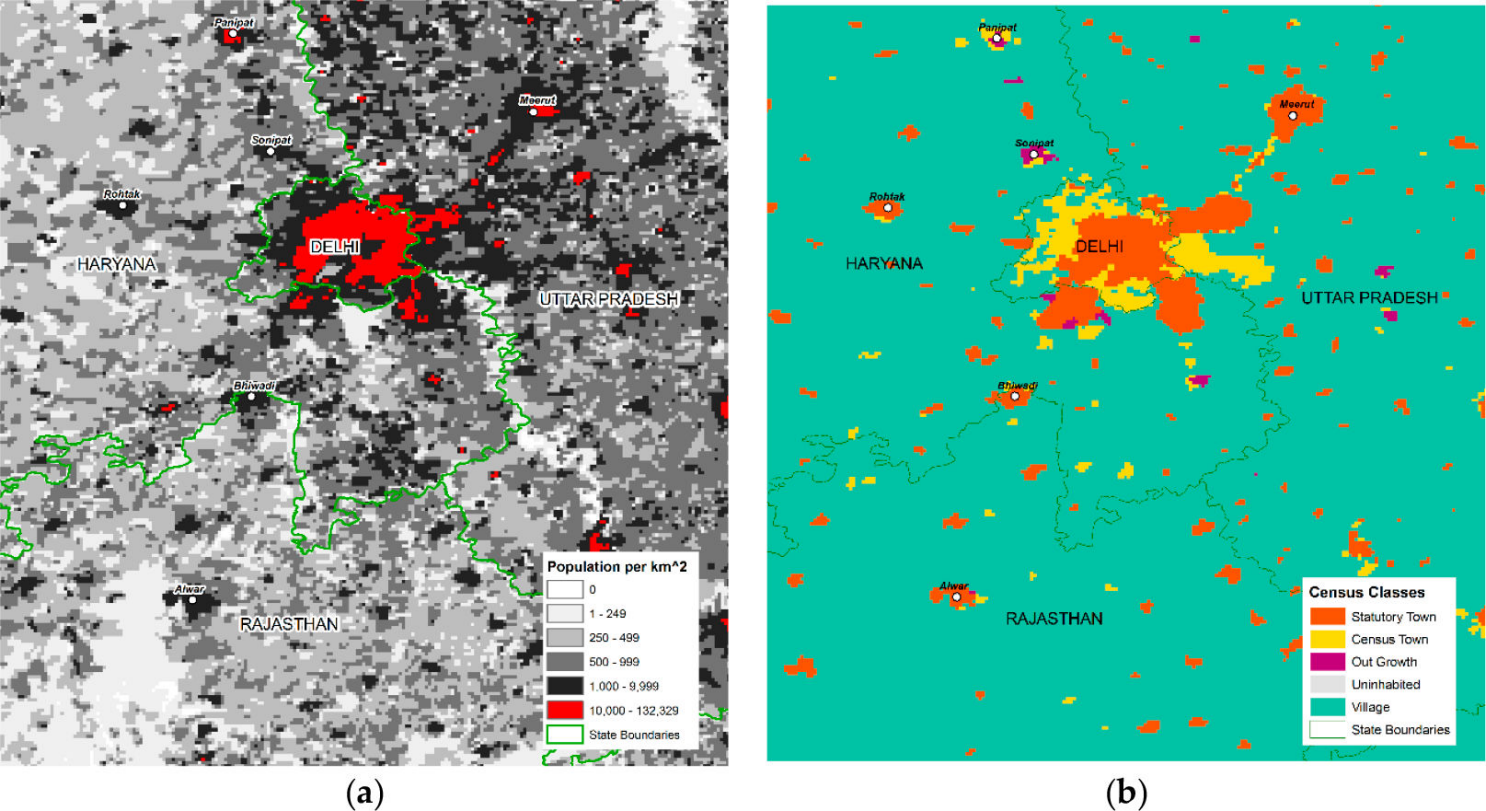

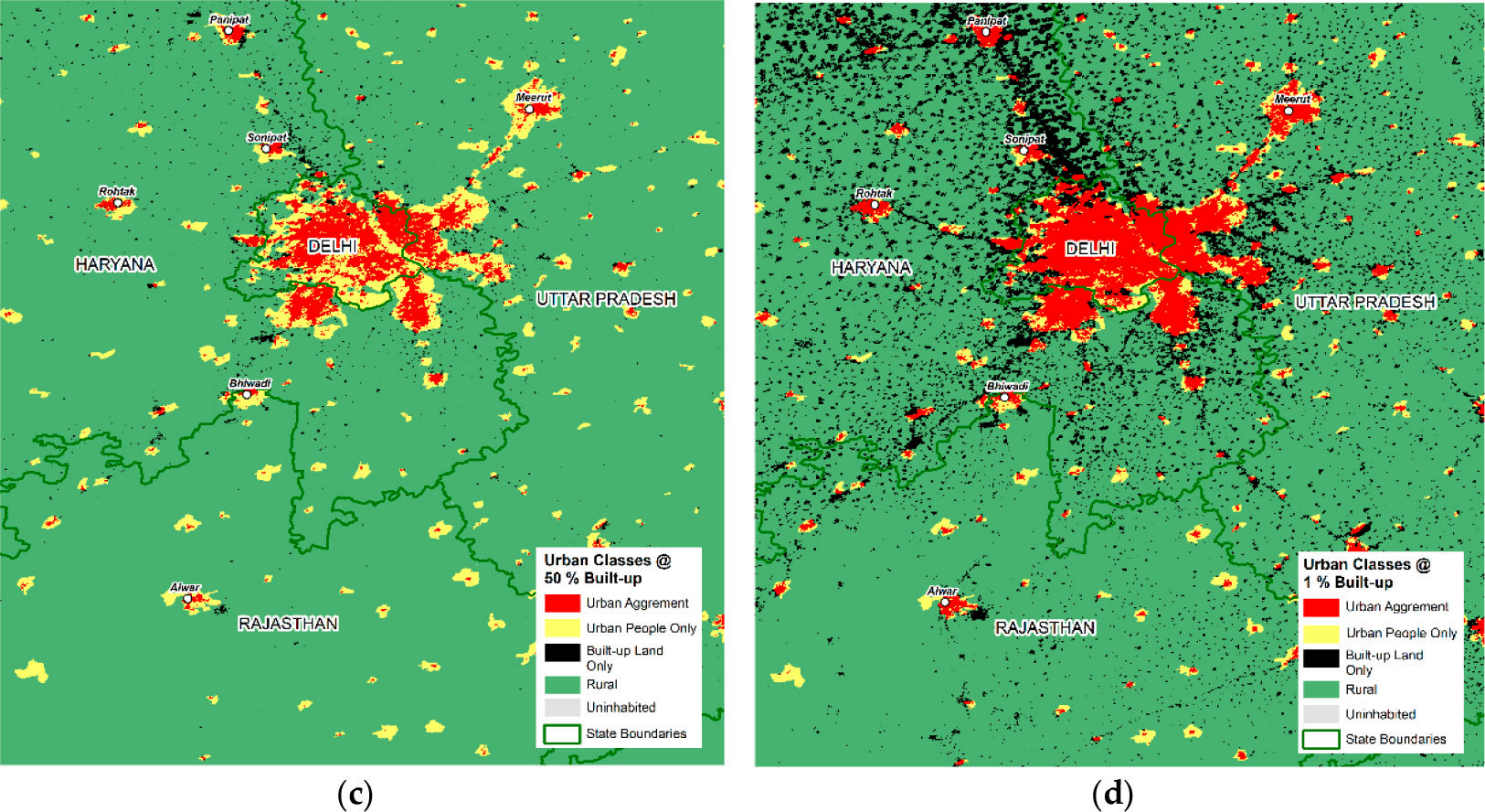
Close-ups of Delhi and surrounding state, 2011: clockwise, the panels indicate (a) population density, (b) *Census Classes*, (c) *Census + GHSL* class at 50% built-up threshold, (d) *Census + GHSL* class at 1% built-up threshold.

Figure 2 shows output data for Delhi and surrounding areas (with labels for selected urban areas). The various panels depict population density alongside two urban classifications: *Census Classes* and *Census + GHSL* (with thresholds of 50 percent and 1 percent built-up shown for comparison). While broad similarities are evident, these three views—particularly in the most densely populated areas—suggest different interpretations of urban India, highlighting especially the situations of smaller urban places whose importance has been emphasized in recent research by Denis and Zerah [25]. To clarify what these maps represent, the methods for generating them are described next.

## 3. Methods

In constructing grids of population and urban areas, we applied a set of common principles to all data production; and for the urban classification grids, some additional methodological considerations were taken into account. The issues are discussed in turn.

### 3.1. Matching Spatial Units with Census Tabulations

As mentioned in Section 2.1.2, modifications to some spatial boundaries were necessary to properly link the detailed PCA census tabulations with the correct spatial units. (As some readers may be aware, the input data from ML Infomap includes population attributes. We did not use those census data in this research, but rather, after validating census codes and adding missing units, particularly for those classified as “outgrowths”, we re-matched the PCA records to the spatial boundaries as described here.) The match of PCAs to the spatial data was made on the following identifiers common to the two databases: (1) state, district, subdistrict (e.g., tehsil), and settlement codes, (2) ward codes where applicable, and (3) outgrowth identifiers. In cases of conflicting information between the PCAs and the spatial information, we gave precedence to the information supplied in the PCAs but checked each potentially problematic match.

One complication in the ML Infomap spatial boundary data is that outgrowths are listed under their rural village identifier codes only. Fortunately, the PCA data on outgrowths include both the urban ward codes and rural village codes, thus enabling a match with the spatial records. A further complication is that in 207 cases, an outgrowth was split between a rural village component and a second component that (although also legally rural) was the part designated as an outgrowth of an adjacent statutory town. Case-by-case inspection showed that a combination of PCA and spatial-data variables could identify each of the two parts of such outgrowths. The PCA records report population and other census information separately for the two components of the divided outgrowths, so there would appear to be no risk of double-counting population and other PCA attributes.

### 3.2. On the Use of Thiessen Polygons

Generally, spatial data on settlement boundaries are available in polygon form. However, some more remote or less populous states have regions in which settlements are represented by points instead of polygons. To create spatially contiguous data that can be gridded, for the point-format settlements we applied a geoprocessing tool in ArcGIS to create Thiessen polygons. These polygons are defined as the area that is closest to each point relative to all other points, and serve as hypothetical boundaries in the absence of official units.

**Figure 3.**
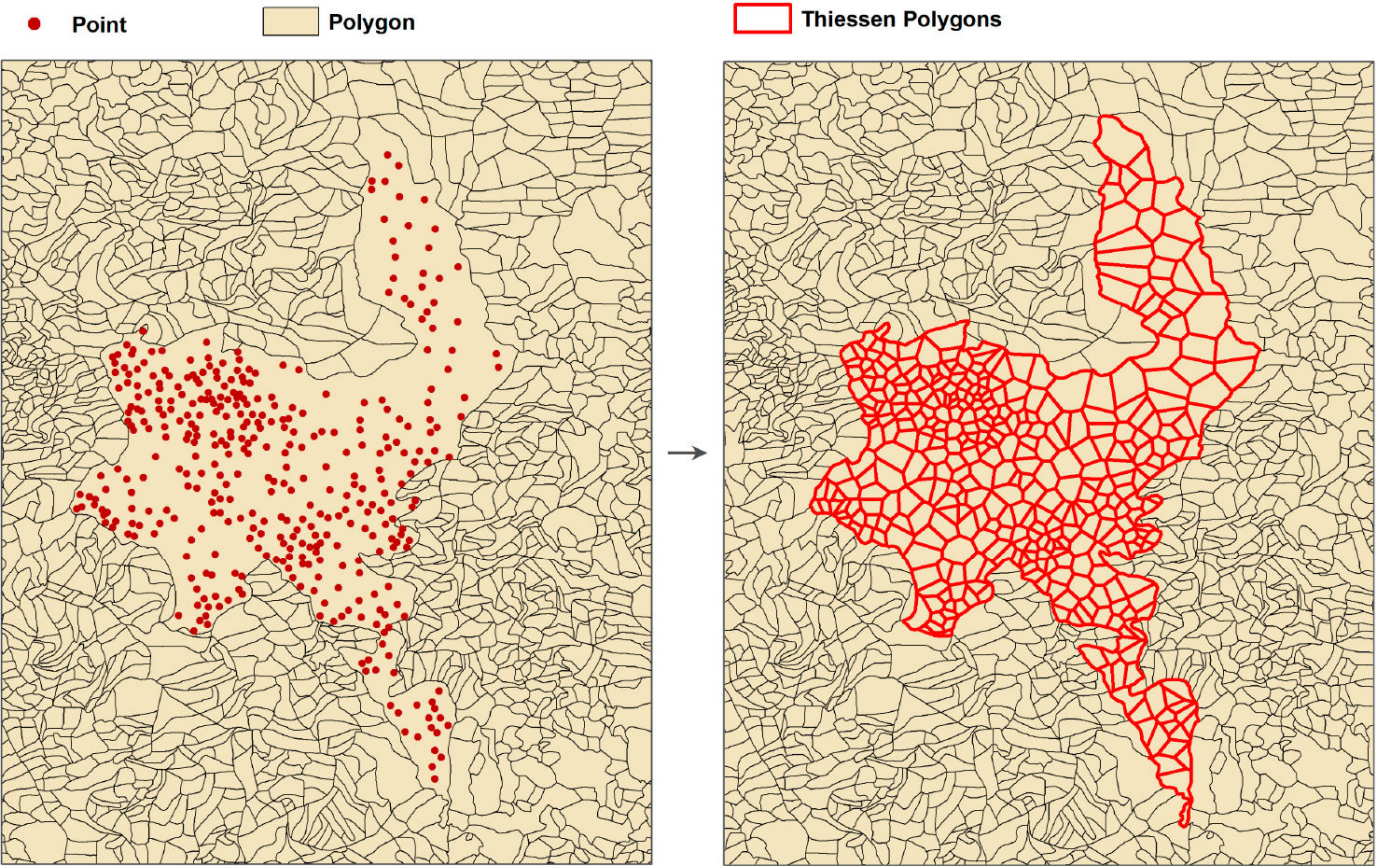
Transformation of point data to Thiessen polygons.

Figure 3 illustrates the transformation from points to Thiessen polygons. For a region in which points represent settlements, the area of the un-subdivided region was allocated according to the placement of the points, producing fictive boundaries around each point. For states with both polygons and points, the Thiessen polygons were always bounded by the surrounding sub-district level boundaries. In areas where many points were provided, we expected a relatively small margin of error. However, for sparse and typically more mountainous areas, some large areas had relatively few settlement points within them, which produced less realistic representations of boundaries. Examples of both types of area are shown in Figure 3.

### 3.3. Transforming Vector Polygons to Raster Grids

**Figure 4.**
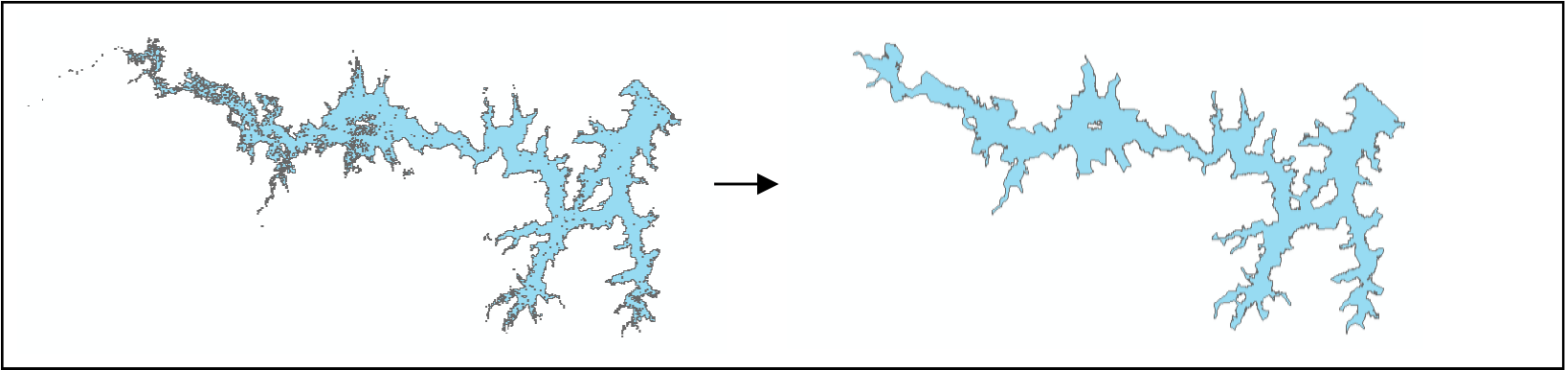
Original and simplified data used as a mask to indicate water (and permanent ice) areas.

Because vector polygons are irregularly shaped, to transform vector data to a uniform grid we used a proportional allocation rule [26] to deal with contributions from multiple areal units to a single grid cell, or from a single areal unit to multiple grid cells. This method is widely used in other gridded population data products [27]. We followed standard practice in removing waterbodies and areas of permanent ice before creating grids of population distribution and urban areas [27]. (The original spatial data from ML Infomap also delineated water bodies in some but not all states, or in some but not all areas within a state. For example, no water is depicted in or around Kolkata, a deltaic city that is located near several major rivers and their tributaries. Due to these inconsistencies, we used a different, more systematic water mask.) The 30 m GHSL data layer indicates major areas of surface water and permanent ice. After simplifying the detailed spatial information in these data (see Figure 4), we used these spatial data as a water mask. The vast majority (about 90%) of the water bodies that were indicated in the ML Infomap data were also detected by GHSL (even if not all water bodies were correctly identified as water by the GHSL). Because water bodies identified in the original ML Infomap spatial data are uninhabited, any waterbody polygon that was not masked by GHSL remains in our collection as an uninhabited polygon.

Data that were originally not in supplied in Geographic World Geodetic System (WGS) 1984 were transformed from their original map projection and all gridding was undertaken in Geographic WGS 1984. Even though the raster units were regular quadrilateral grids, due to the Earth’s curvature the land area of a 30” (nominally, 1 km) grid cell at the southern tip of India is greater than it is at the northern tip. For this reason, it is necessary to construct a measure of population density rather than population counts. For this, area grids are necessary. Two area grids accompany this data collection:

- A land area grid which indicates the total land area in each grid cell. (As noted above, water bodies are removed).
- A land area grid that indicates the land area of a grid cell in a given Indian state. This allows for the land area of border zones (as well as in coastal areas) to be treated fractionally.

**Figure 5.**
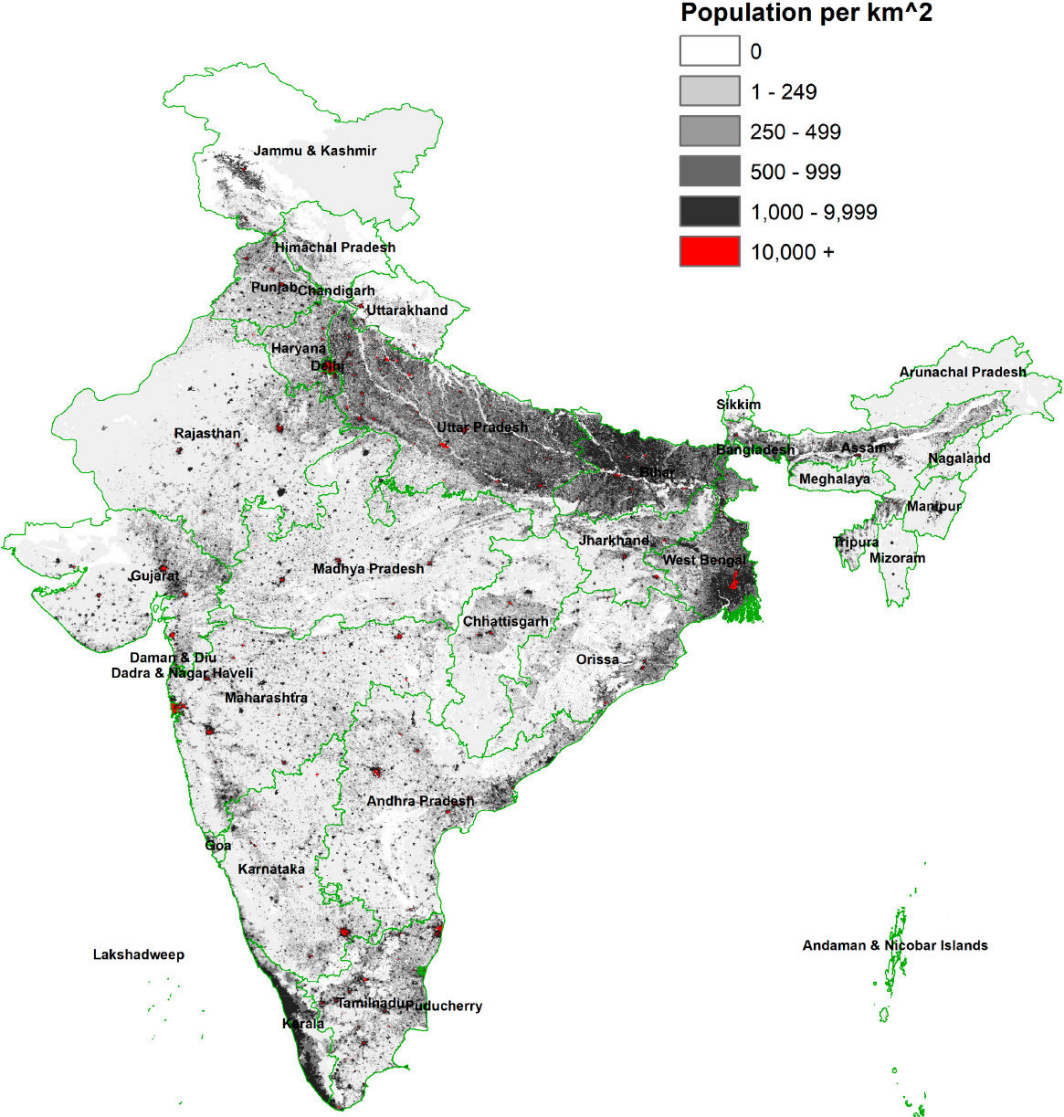
Population density, India 2011.

In Figure S1, a flow diagram depicts the processes used to transform vector to raster data from census data alone. Note the two variants used to construct the population grids and urban classification *Census Classes* grids. The processes used to generate the urban *Census + GHSL* grids is laid out in Balk et al. [18] with the modification that the census units are from the Indian census rather than the US census. Additional details for the urban grids are described below. The resulting population grid is shown in Figure 5.

### 3.4. Construction of Urban Classes

We constructed two urban classification grids, one based exclusively on census information and the other produced by integrating the census classifications with GHSL data.

#### 3.4.1. Census-Only Grids

**Figure 6.**
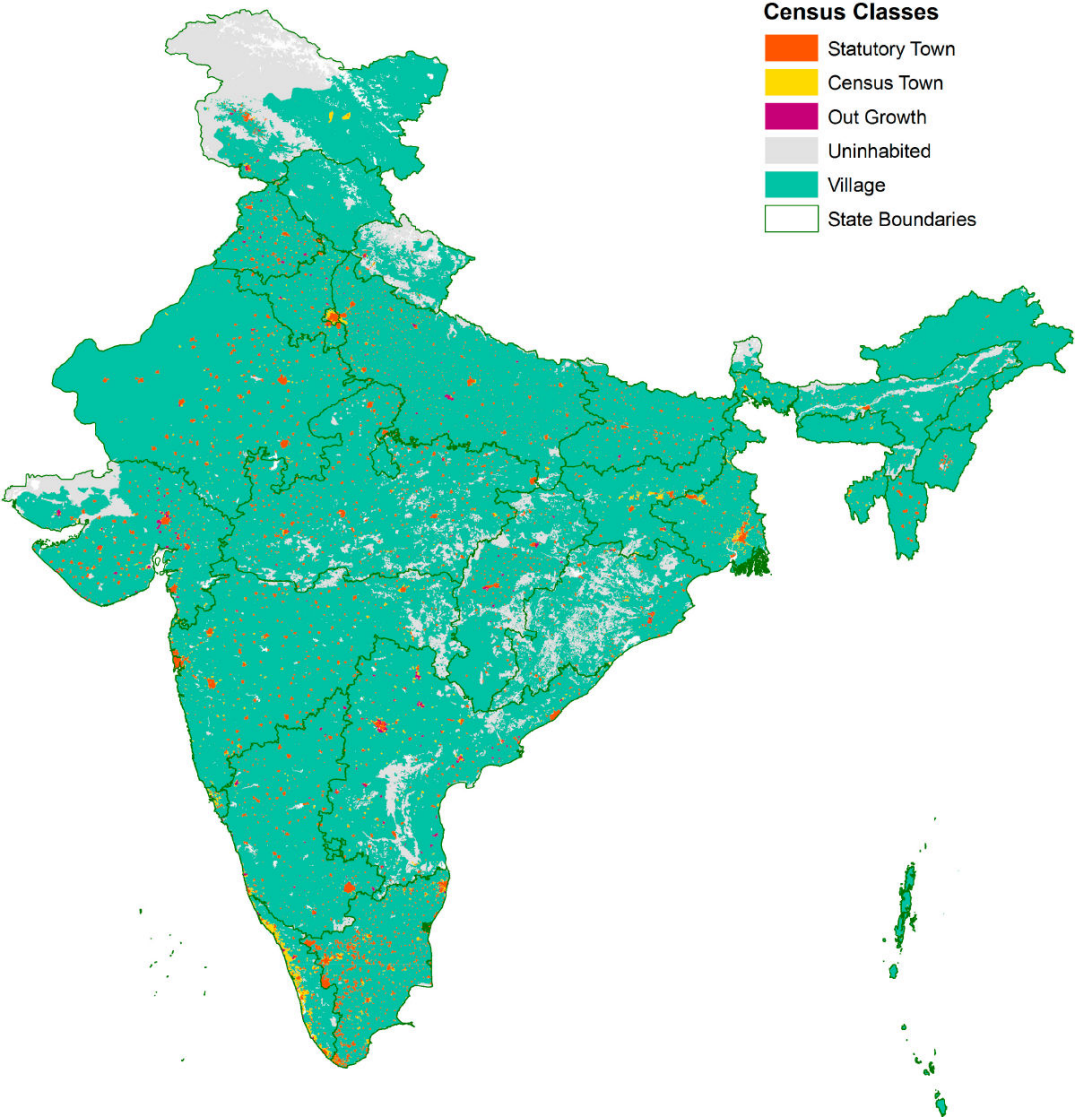
Urban classifications based on census classes, India 2011.

The first classification grid, which we denoted as *Census Class*, is based only on the official census categorization of settlements. As discussed earlier, these categories include statutory towns (STs), outgrowths (OGs), census towns (CTs), and rural villages. Wards which are part of a statutory town for which we had ward-level spatial data, are indicated as components of a statutory town. The census classification was transformed from polygon to grid format using a majority rule: the classification indicated in the grid cell is the majority class of the input vector polygons. For example, a cell comprised of 51% of ST units (by area) and 49% of CT units would be classified as ST. If multiple settlement classes overlay a cell—usually something that occurs on the outskirts of urban areas—the maximum contributor was assigned the classification even if the value was less than the majority of the cell’s area. The resulting grid for all India is shown in Figure 6.

A summary of the *Census Class* grid is presented in Table 3. Corresponding to the census’s national urban estimates, 31% of India’s population lives in one of the three urban settlement types. Twenty-six percent of the population lives in areas classified as statutory towns, which make up 2.5% of India’s land area. Census towns and outgrowths occupy far less area, and are home to much less of India’s urban population, but outgrowths (which occupy less than 0.1% of all land area) are home to more than 4 million persons. Average population densities for these three classes indicate a continuum of urban locations: notably, outgrowths have population densities above 1200 persons/km^2^, more than four times the average for villages.

To demonstrate the utility of these spatial data, and to anticipate results from our combined census–GHSL grid, Table 3 also shows the GHSL built-up percentage of each type of urban area. To estimate these built-up percentages, we overlay the census data layer with the GHSL built-up raster and compute the average value for each census class. As with population density, a clear gradient is seen across the classes. We find that although outgrowths are legally rural, they exhibit built-up densities characteristic of statutory towns and census towns.

#### 3.4.2. Census and GHSL-Based Classification

The second classification grid integrates the official census classifications with the GHSL built-up layer, producing an urban/rural schema that we have described in detail elsewhere [18]. The general idea is to create a two-way classification of land, with one dimension reflecting the official urban and rural definitions and the other summarizing the built-up area estimate from GHSL. For the official classifications, we treat a location as urban if it is classified as an ST, CT or OG, and otherwise take it to be rural or uninhabited, if designated as such in the boundary data (see Table 1). For the built-up land layer, we similarly adopt a binary measure based on thresholds of built-up proportions in each raster cell, classifying each cell as either built-up or not built-up according to its density relative to the chosen threshold. Two thresholds were considered here: a 50% built-up threshold, which is the most commonly-cited threshold for defining urban land from GHSL [21] and a 1% threshold, for greater inclusivity [19].

This combination yields four classes: (1) a class of *urban agreement* (denoted as UAg), in which an area is officially classified as urban and also exceeds the GHSL density threshold; (2) a class of area we denote as *urban people only* (UPO) for areas that are officially urban but which fall short of the GHSL threshold; (3) a class of *built-up land only* (BULO) for areas that are not officially urban but which nevertheless exceed the density threshold; (4) a *rural extent class* (RE) for land that is neither officially urban nor sufficiently built-up; and (5) because the boundary data identified *uninhabited areas*, we constructed a separate class for that as well. We integrated the resulting four classes of UAg, UPO, BULO, and RE (along with uninhabited) to produce a single grid.

**Figure 7.**
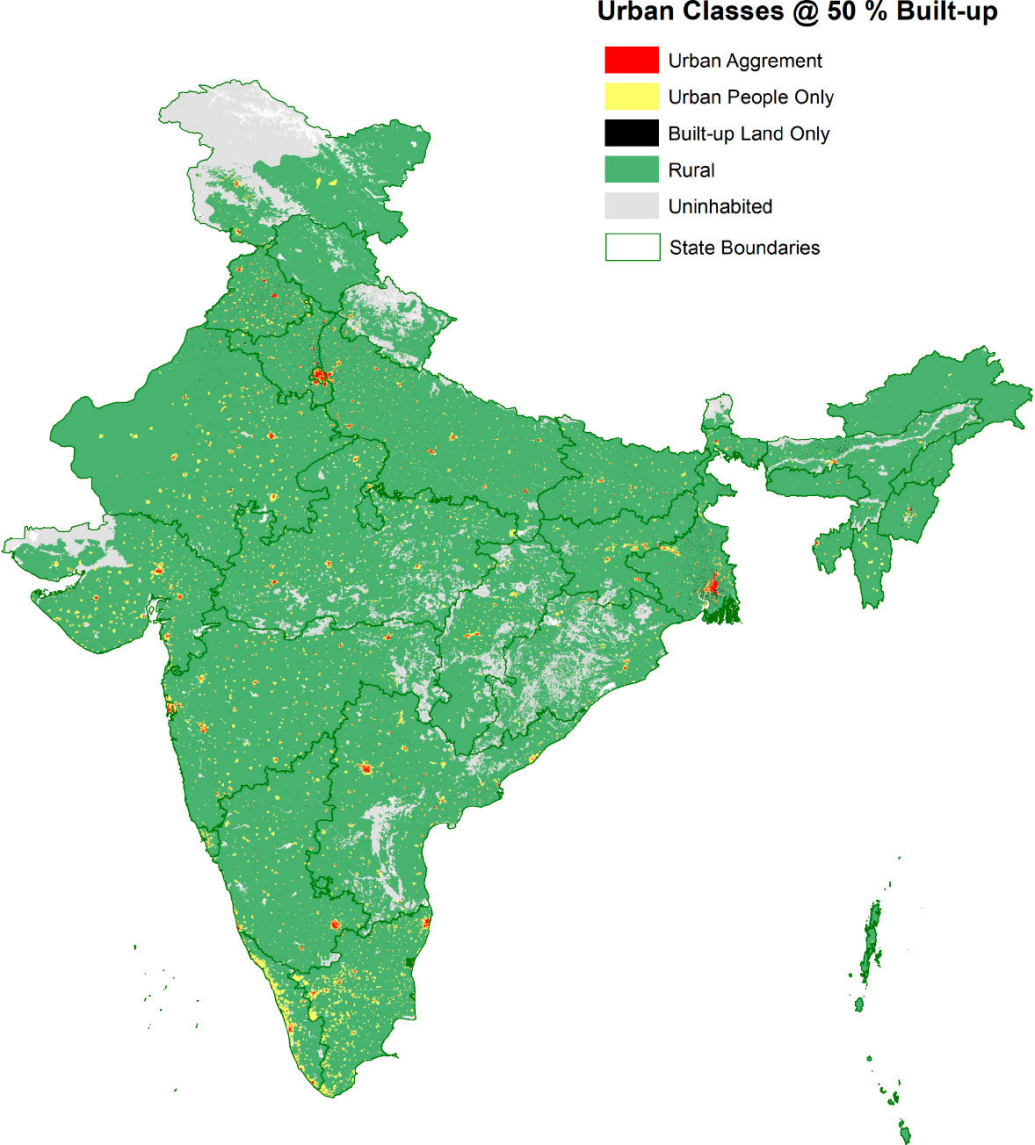
Urban classifications based on census classes combined with GHSL 50% built-up, India 2011.

To carry out this process, the high-resolution (250 m) GHSL data were vectorized and overlaid with the census vector data. The final product remains in vector format and can be rendered to a raster grid at the same 250 m resolution (users may coarsen it to 1 km to match the other grids, and in all cases will have to decide how to treat mixed-class cells when rasterizing.) The results are presented in Figure 7.

A summary of these classes (using both the 50% and 1% built-up thresholds), which shows the population, land area, and the average built-up fraction, is given in Table 4. As the table shows, areas that are both officially urban and sufficiently built-up, that is, areas of *urban agreement* (UAg), are home to 10.8 percent of India’s population but account for only 0.4% of its land area. These urban-agreement areas, which might be regarded as core-urban locations, are more than 75% built-up, a level well above the 50% definitional threshold. Their average population density exceeds 10,000 persons/km^2^, more than 2.5 times the statutory town average population density. In contrast, about 1 in 5 persons in India lives in an area that is officially urban but which is less than 50% built-up; yet such UPOs areas occupy much more land than do the areas of urban agreement. By definition, UPO areas fall short of the 50% GHSL threshold, but the degree to which they do is striking: they are less than 5% built-up on average. Nevertheless, these areas have high population densities, more than 2500 persons/km^2^. Finally, areas that are built-up but not officially classified as urban (BULO) are home to only 5.5 million residents across India. Although not as dense as UAg or UPO areas, the population densities in these areas exceed 1000 persons/km^2^. The presence of BULO areas varies considerably across states, supporting recent research findings that indicate considerable state-specific variation in urbanization (perhaps due to state-specific incentives or development initiatives); in some states, urbanization is both widespread and decentralized, occurring outside major cities [25].

Table 4 and Figures 2 and 5, and Figure S2 reveal that these classifications are sensitive to the built-up threshold.

As Tables 3 and 4, and Figures 6 and 7 show, the two different urban classes reveal different aspects of urbanization. The availability of both official and remotely-sensed measures will enable a wide community of users to examine India’s complex patterns of urbanization in fine-grained spatial detail.

## 4. User Notes

These data were constructed to nest into many of the more commonly used global gridded data products at a 1-km resolution (such as the Gridded Population of the World (GPW) version 4 [28]). The data also nest directly into existing gridded projections of future population under scenarios such as the Shared Socioeconomic Pathways (SSPs) [29] when aggregated to 7.5′ resolution [30]. In the case of the latter, the improvements over existing Indian spatial population data offered by this product, as well as the new, spatially explicit information contained in urban classification grids, may improve the ability of the research community to produce scenario-based projections of population/urbanization outcomes for India, a country of substantial importance in the global-change community.

## Supplementary Material

India_Supplement

## Figures and Tables

**Table 1.** Overview of input vector spatial data, matched with Primary Census Abstracts: Number of Spatial Units by Format and Urban Classification Type.

States & Union Territories	Format		Urban Classification *				Village

	Polygon	Point	Statutory Town	Census Town	Ward	Outgrowth	

Andamans & Nicobars	-	560	1	4	-	-	555
Andhra Pradesh	26,927	2090	125	228	287	209	27,800
Arunachal Pradesh	-	5616	26	1	-	-	5589
Assam	22,746	4137	88	126	89	29	26,395
Bihar	45,159	0	139	60	76	4	44,874
Chandigarh	38	0	1	5	28	2	5
Chhattisgarh	18,528	2255	168	14	110	40	20,126
Dadra & Nagar Haveli	71	0	1	5	-	-	65
Daman & Diu	27	0	2	6	-	-	19
Delhi	256	0	3	110	-	-	112
Goa	414	0	14	56	7	7	334
Gujarat	19,040	0	195	153	377	127	18,225
Haryana	7080	0	80	74	83	15	6841
Himachal Pradesh	13,103	7693	56	3	8	8	20,689
Jammu & Kashmir	6766	241	86	36	232	93	6553
Jharkhand	32,884	0	40	188	111	1	32,394
Karnataka	30,232	0	220	127	459	69	29,340
Kerala	1871	0	59	461	173	16	1018
Lakshadweep	-	27	-	6	-	-	21
Madhya Pradesh	56,346	0	364	112	295	86	54,903
Maharashtra	45,926	0	256	278	898	3	43,665
Manipur	493	2170	28	23	7	7	2582
Meghalaya	-	6861	10	12	-	-	6839
Mizoram	-	853	23	-	-	-	830
Nagaland	-	1454	19	7	-	-	1428
Odisha	53,283	0	107	116	171	57	51,311
Puducherry	101	0	6	4	1	1	90
Punjab	13,055	0	143	74	261	61	12,581
Rajasthan	45,287	0	185	112	291	39	44,672
Sikkim	484	0	8	1	-	-	451
Tamil Nadu	17,450	0	721	376	373	14	15,979
Tripura	917	0	16	26	-		875
Uttar Pradesh	108,336	0	648	267	593	63	106,774
Uttarakhand	16,835	293	74	41	49	19	16,793
West Bengal	41,482	0	129	781	286	13	40,202

Total	625,137	34,250	4041	3893	5265	983	640,930

* Unpopulated spatial units are indicated by a variety of land-use types (e.g., forest, submerged areas, mountain) and are not indicated here. Totals for the urban classes are not mutually exclusive: for a single statutory town with many ward-level spatial units, the counts here include both the respective ST and wards totals. Wards also include outgrowths (available for all statutory towns and cities), which are also noted in separate column above, as well as “regular” wards (available only for selected large cities as shown in Figure 1).

**Table 2.** Overview of key datasets on urbanization in India, 2011.

Theme	Data File	Concept	Format (Resolution)	Type	Values
**Population Counts**	Pop	De jure population as indicated by the census	Raster (1 km)	Integer	0–136,626 persons ^1^
**Area**	Area ^2^	Actual land area of each grid cell	Raster (1 km)	Integer	
	Area, delineating border cells	Actual land area of each grid cell delineating border cell (e.g., coastline, between states)	Raster (1 km)	Integer	
**Urban Classifications**	*Census Classes*	Census designations of settlement type	Raster	Categorical	Statutory Town, Census Town, Outgrowth, Village, Uninhabited
	*Census + GHSL*	Census designations of settlement type combined with built-up area thresholds ^3^	Vector (based on 250 m raster and variable resolution vector inputs)	Categorical	Urban Agreement (UA), Urban People Only (UPO), Built-up Land Only (BULO), Rural Extents, Uninhabited

1 There are 14 1-km grid cells in India with a population count greater than 100,000 persons. All are found within urban areas in the following states: Delhi (3), Gujarat (3), Madhya Pradesh (1), Maharashtra (5), West Bengal (2).

2 Note that the area grid is supplied as one all-India grid rather than state-specific grids.

3 This data layer was produced and disseminated using two built-up area thresholds from the Global Human Settlement Layer (GHSL): 50% and 1%.

**Table 3.** Population, area, and population density (2011), and estimates of percentage built-up (2014), by official census classifications, India.

Census Classification	Population		Area		Population Density	Built-Up
	
	Count	%	km^2^	%		%

Statutory Town	318,562,520	26.3%	80,109	2.5%	3977	14.4
Census Town	54,280,980	4.5%	26,234	0.8%	2069	10.2
Outgrowth	4,264,979	0.4%	3436	0.1%	1241	8.7
Village	833,746,498	68.9%	2,850,979	87.2%	292	0.6
Uninhabited	0	0.0%	307,377	9.4%	-	0.1

NB: These summaries were produced from the underlying vector data. Statistics based on the gridded products will differ slightly due to rounding and the majority-rule assignment of each class to a given grid cell.

**Table 4.** Population, area, and population density (2011), and estimates of percentage built-up (2014), according to urban classification based on *Census + GHSL* (satellite-derived) built-up area data, India.

Threshold	Urban Classification	Population		Area		Population Density	Built-Up
	
		Count	%	km^2^	%		%

50	Urban Agreement (UAg)	130,203,192	10.8%	12,569	0.4%	10,359	78.3
	Urban People Only (UPO)	246,902,934	20.4%	96,238	3.0%	2566	4.7
	Built-Up Land Only (BULO)	5,554,092	0.5%	5061	0.2%	1097	66.8
	Rural Extent (RE)	828,194,760	68.4%	2,830,261	87.5%	293	0.4
	Uninhabited	-		290,373	9.0%		0.1

1	Urban Agreement (UAg)	241,523,146	19.9%	40,706	1.3%	5933	35.3
	Urban People Only (UPO)	135,582,980	11.2%	68,101	2.1%	1991	0.0
	Built-Up Land Only (BULO)	86,191,197	7.1%	140,894	4.4%	612	11.3
	Rural Extent (RE)	747,557,654	61.7%	2,697,763	83.4%	277	0.0
	Uninhabited	-		287,038	8.9%		0.0
