# Supplementary figures and images for: Urbanization in India: Population and Urban Classification Grids for 2011
Source: Data (Basel). Author manuscript; Available in PMC 2023 Jul 7. (PMC10327898; doi:10.3390/data4010035)

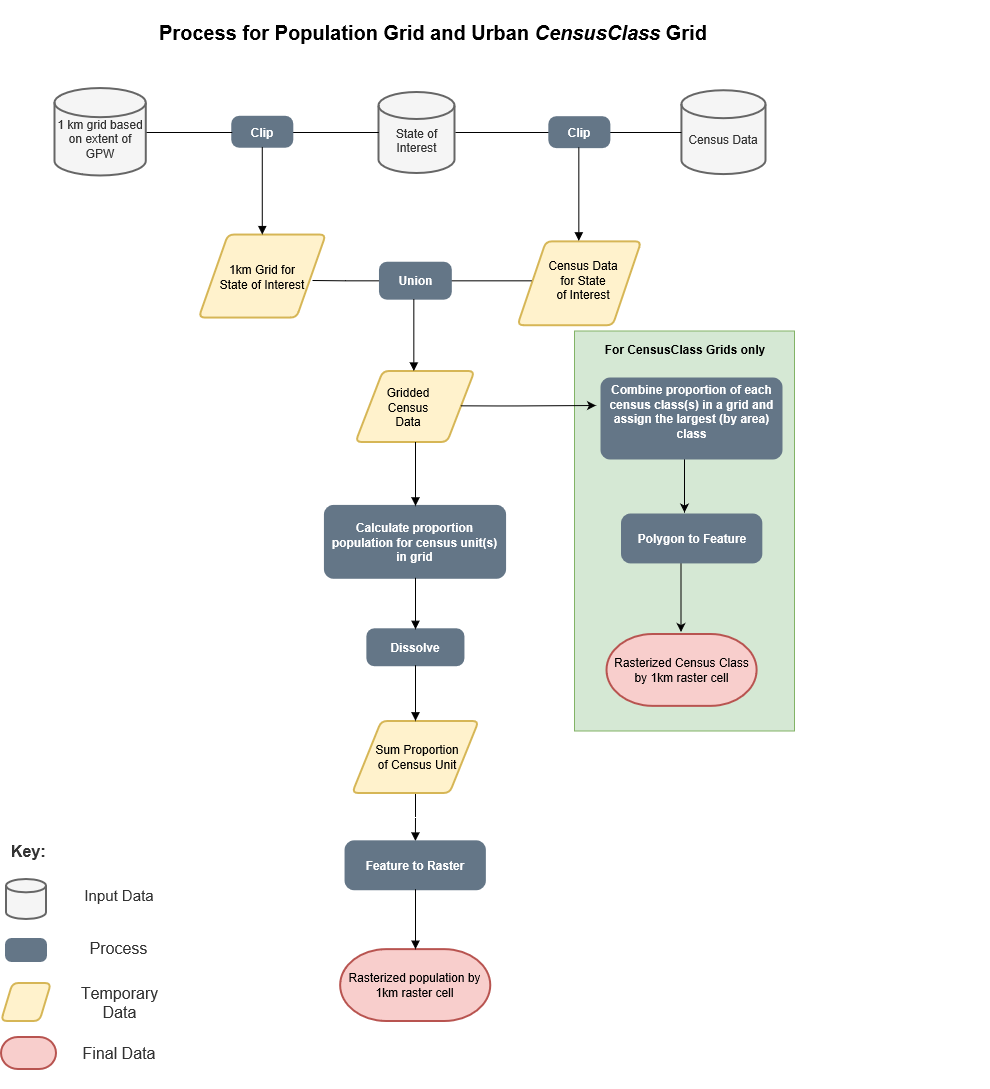

Supplement: India_Supplement [file NIHMS1770657-supplement-India_Supplement.zip › Figure_S1_FlowChart.png]

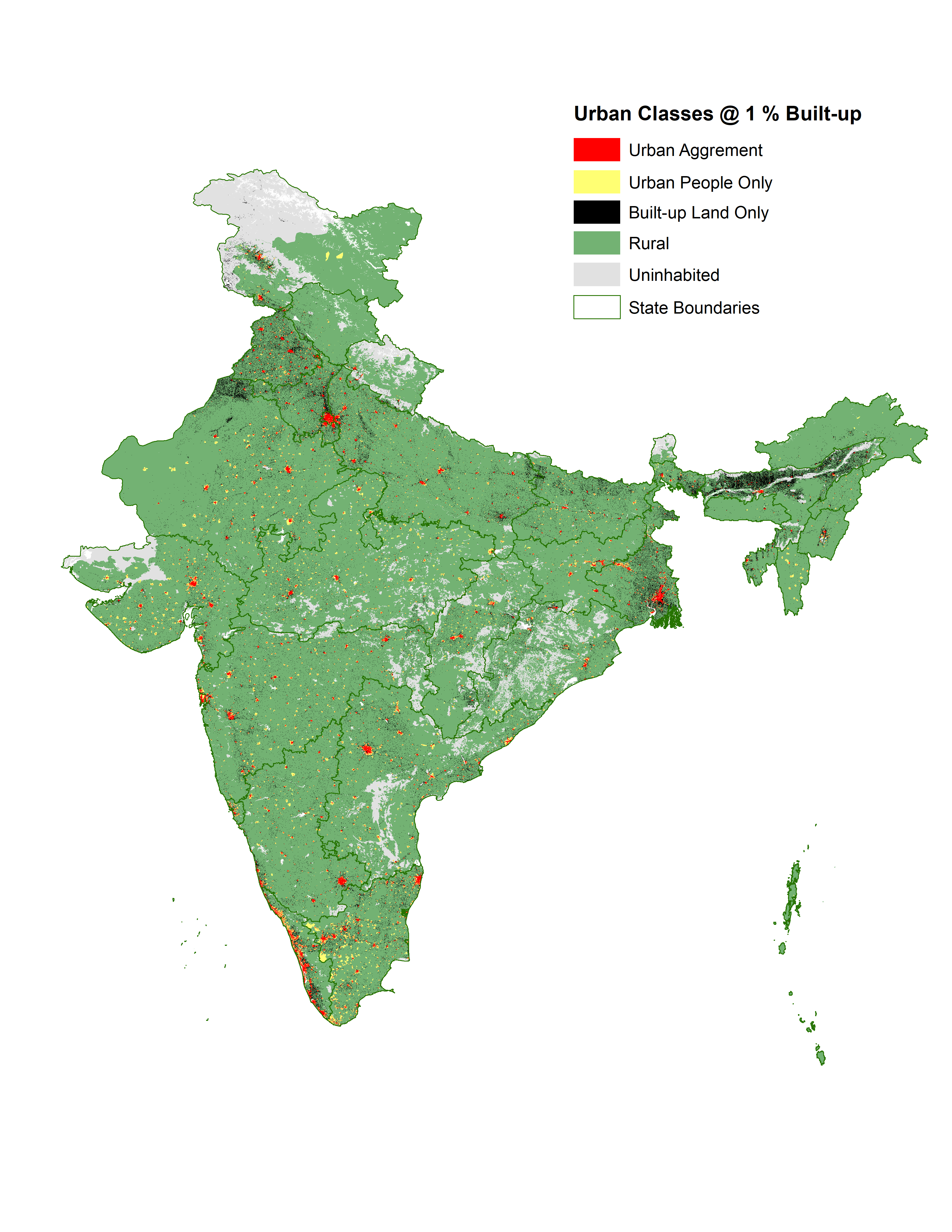

Supplement: India_Supplement [file NIHMS1770657-supplement-India_Supplement.zip › Figure_S2_india_census_ghsl_01pct.tif]
